# One Health Perspectives on New Emerging Viral Diseases in African Wild Great Apes

**DOI:** 10.3390/pathogens10101283

**Published:** 2021-10-06

**Authors:** Denis S. Azevedo, José Lucas C. Duarte, Carlos Felipe G. Freitas, Karoline L. Soares, Mônica S. Sousa, Eduardo Sérgio S. Sousa, Ricardo B. Lucena

**Affiliations:** 1 Veterinary Pathology Laboratory, Agrarian Science Center, Federal University of Paraíba, Areia 58397-000, PB, Brazil; denis.azevedo@academico.ufpb.br (D.S.A.); jlcd@academico.ufpb.br (J.L.C.D.); carlos.freitas2@academico.ufpb.br (C.F.G.F.); karoline.lacerda@academico.ufpb.br (K.L.S.); 2 Graduate Program in Animal Science and Health, Rural Health and Technology Center, Federal University of Campina Grande, Avenida Universitária, S/N Jatobá, Patos 58708-110, PB, Brazil; monica.shinneider@estudante.ufcg.edu.br; 3 LaBiMol, Medical Science Center, Federal University of Paraíba, João Pessoa 58051-900, PB, Brazil; esergiosousa@uol.com.br

**Keywords:** zoonosis, zooanthroponosis, infection, disease, respiratory, primate, simian

## Abstract

The most recent emerging infectious diseases originated in animals, mainly in wildlife reservoirs. Mutations and recombination events mediate pathogen jumps between host species. The close phylogenetic relationship between humans and non-human primates allows the transmission of pathogens between these species. These pathogens cause severe impacts on public health and impair the conservation of habituated or non-habituated wild-living apes. Constant exposure of great apes to human actions such as hunting, deforestation, the opening of roads, and tourism, for example, contributes to increased interaction between humans and great apes. In spite of several studies emphasizing the risks of pathogen transmission between animals and humans, outbreaks of the reverse transmission of infectious agents threatening wildlife still occur on the African continent. In this context, measures to prevent the emergence of new diseases and conservation of primate species must be based on the One Health concept; that is, they must also ensure the monitoring of the environment and involve political and social aspects. In this article, we review and discuss the anthropological aspects of the transmission of diseases between people and wild primates and discuss new anthropozoonotic diseases in great apes in Africa from studies published between 2016 and 2020. We conclude that the health of great apes also depends on monitoring the health of human populations that interact with these individuals.

## 1. Introduction

The One Health concept proposes the inseparability between human, animal, and environmental health in a cooperative, optimistic, and adaptive approach to achieving health in a disturbed biosphere [1,2]. In this context, pathogens that affect great apes must be investigated in association with the environmental context and the relations with the people of a particular region, country, or even the continent. Thus, measures to prevent outbreaks and spread of existing diseases or the emergence of new diseases, and promote the conservation of species, must also guarantee environmental monitoring and encompass political and social aspects. 

On the African continent, many socio-political factors make animal and human populations vulnerable to zoonotic diseases. Zoonotic episodes reported in Africa show that social differences significantly impact health risks in different countries [3]. It is proven that, for being marginalized, the poorest people are often exposed to the most adverse conditions. They cultivate, herd, hunt, sell, or work in the most exposed places, have less protected housing infrastructure, and cannot benefit from preventive or protective measures. On top of this, marginalized people have limited political capacity and voice to make claims to the state to mitigate and manage disease risks [4,5]. 

In Africa, social inequality and its resulting impacts are impediments to national and human development. Thus, risk factors tend to be perpetuated in African countries [6]. A vicious circle is formed between rural poverty and environmental degradation: environmental degradation contributes to poverty by worsening the health of poor people and restricting the productivity of the resources on which they depend, while poverty pushes poor people to interact in harmful ways with the environment [7]. Thus, the condition of poverty of these individuals is caused by environmental problems and contributes to environmental degradation.

In addition to environmental degradation, new relationships between humans and wild animals, especially non-human primates, have increased the exposure of a much larger population, including people living in urban areas, to health risks. These new interactions range from deforestation for the construction of new roads or expansion of agricultural activities to the contact of these animals, or potential vectors of disease, with people who travel in search of ecotourism opportunities [8]. It is important to note that human actions pose the risks both of pathogen transmission among humans and pathogen transmission from humans to great apes. This review summarizes the current knowledge about the anthropological aspects of the relationship between non-human primates (NHPs) and humans in recent episodes of disease transmission to wild great apes on the African continent.

## 2. Anthropological and Behavioral Aspects in the Occurrence of Emerging Diseases in Great Apes

People and their environments maintain an inseparable relationship, and thus the practices of a given community are circumscribed within the socio-environmental context in which they exist. This interaction between humans and the environment determines disease patterns as they appear in the population. Despite gaining greater attention in recent years, this concept dates back to Hippocrates and was coined as “One Medicine” by Schwabe [9]. It is evident that the environment represents the geographical space and the ecological position that humans hold, taking into account the individual and community identities, ecological flows, and social relationships [10].

**Figure 1:**
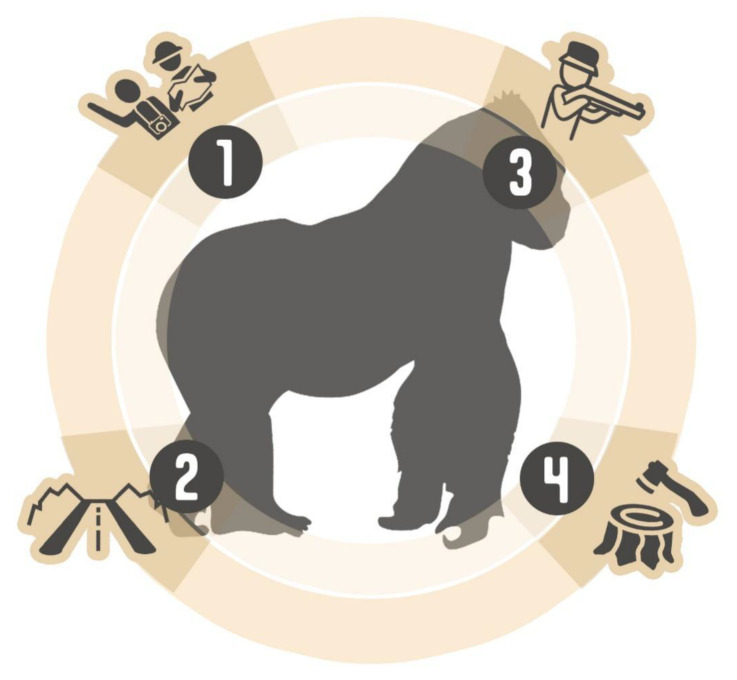
The increase of human–animal–environment interdependence may be the most critical risk factor to non-human primates’ health and well-being in relation to infectious diseases. These include (**1**) tourism; (**2**) asphalt road construction; (**3**) wildlife hunting (i.e., “bushmeat”); and (**4**) deforestation.

Understanding social issues clarifies that health and disease are based on dimensions beyond biological sciences for humans and animals (Figure 1). Therefore, health sciences, including veterinary sciences, must be in close association with social sciences and anthropology, in a biocultural approach to the issue [9,10,11,12,13,14,15]. For example, medical anthropology is, roughly speaking, a field that investigates health and disease factors, taking into account the ways of life of different populations [10]. The ways in which individuals relate to the environment in which they live occur as part of the social context that contains them. These aspects should be considered in investigations of emerging pathogens, as established, for example, in studies that determined the origin of the human immunodeficiency virus (HIV) [16]. It is known now that retroviruses in NHPs are related to HIV in humans; that is, that simian immunodeficiency virus evolved into HIV [17]. Investigations concluded that this virus originated in chimpanzees and gorillas from West Central Africa [18] and some other studies have also shown a relationship with NHPs from East and West Africa [19]. This cross-infection possibly resulted from the predation of NHPs (i.e., “bushmeat” hunting) by impoverished populations in food and nutritional insecurity [20]. 

Because some populations are at a greater likelihood of exposure to certain diseases, such as zoonoses, due to their settlement in specific socio-ecological environments [21], we must understand the socio-economic vulnerabilities, socio-political factors, and cultural inheritance that cause risks to rise [14,22]. The Ebola outbreaks that spread to different African communities and countries every decade perfectly exemplify the importance of this complex social, political, and ecological relationship in the dynamics of the occurrence and persistence of an emerging disease. Among the emerging pathogens, viruses of the Filoviridae family, responsible for hemorrhagic fevers, cause severe problems in public health and the conservation of species in Africa [23]. These agents were first diagnosed over 40 years ago, usually affecting rural populations, but since then, they have caused absolute terror in African countries due to their high transmissibility and lethality, crossing borders, and recently acting on the outskirts of large cities [24]. In many human outbreaks, the search for the index case confirmed the occurrence of direct contact with the carcass or blood of infected primates [23]; however, the form of transmission to the NHPs remains obscure [25]. 

The high percentage of people living in rural areas who depend on livestock production [26] or hunting [27] to survive is one aspect that explains why poor rural people are more likely to be exposed to endemic zoonoses. In addition, there is very close contact with animals in these areas, which favors the triggering of outbreaks of zoonoses or anthropozoonoses. Furthermore, the United Nations estimates that, in 2020, the rural population of Africa would account for 56.2% of the continent’s total population [28], further increasing the abovementioned risks. Therefore, we can predict that poverty/exclusion, subsistence, and high rural density are factors that will altogether prompt the emergence of new infectious diseases. However, the relationship between people, animals, and the environment is very complex.

Understanding the transmission of zoonotic diseases between wild animals and humans on the African continent must be understood as involving various factors. Among them are recurring political crises and subsequent social insecurity that results in a continuous movement of people towards exploiting natural resources [29]. In addition, the intensification of subsistence agriculture in many countries increases the extent of human invasion of wildlife habitats. Hunting, butchering of carcasses, and subsequent consumption of meat from wild animals are also significant risk factors for the most impoverished populations [30] since this type of food in part contributes to food security for poor rural families facing chronic malnutrition. Under these conditions, hunting and consumption of wild animals increase when alternative livelihoods collapse [31].

However, hunting of African wildlife is not limited to conditions of subsistence. Some variables were brought up in wildlife use surveys in four African countries (Ghana, Tanzania, Madagascar, and Cameroon) [31]. The data considered the geographical and economic position of more impoverished rural communities and the wealthy urban class in a relationship between hunting, market, and bushmeat consumption. It was found that not only the poorest people in rural areas consume bushmeat in these areas, but the wealthier urban class also does. The following factors were considered to contribute to this finding: the proximity of the urban area to the source of capture, leading to lower prices; lower prices than alternative food; the proximity of hunters to urban markets, which led them to sell up to 80% of their catch to people outside the rural community, while 75% to 90% of wildlife harvested in the most isolated settlements was consumed locally by the hunter’s household or neighbor; as bushmeat price increases, due to the difficulties imposed by the distance and time spent hunting to hunters, wealthier people continue to consume it because they have greater purchasing power. This shows that zoonotic diseases’ risks have become universal in these countries and are not limited to isolated populations. 

In addition to the factors described above, new pathogen transmission dynamics between people and apes have been identified. This is even more complex, as it involves the movement of people from distant regions. Despite the fact that the habituation of great apes and their contact with people is related to the transmission of pathogens, human intervention has contributed to increasing ape populations. This is because veterinary care and awareness of local populations have allowed for better conservation of these groups of primates [8]. Furthermore, in some parts of sub-Saharan Africa, chimpanzee tracking is a popular tourist activity, offering visitors the chance to see chimpanzees and other primate species in their natural habitats [32]. Animal encounter experiences provide extraordinary moments that can change the lives of human participants and turn them into advocates of the species they encountered [33]. Furthermore, ecotourism can be an essential source of revenue that benefits the conservation of the species [33]. Therefore, applying guidelines for sustainable tourism based on a One Health approach, including mandatory vaccination and testing of visitors and residents, will prevent human to great ape pathogen transmission [8,34]. 

## 3. New Diseases Transmitted from Humans to Great Apes

Zooanthroponoses are diseases that are transmissible from human to human that can also be transmitted from people to animals; such diseases are also referred to as anthroponotic. Many of these pathogens evolved through different animal species until reaching NHPs and humans. Infection by human pathogens in primates has been related to close contact with humans diagnosed with viral infection [34]. In recent years, more and more infectious agents have acquired this ability to cross inter-species barriers (Table 1). One such agent is SARS-CoV-2, which apparently originated in bats, but adapted to other host species, including humans, and caused the COVID-19 pandemic [35]. In African apes, important diseases can be transmitted through respiratory droplets, fecal–oral contamination, skin contact, bites, or by an arthropod vector [21,34].

In the same way that pathogens are transmitted from great apes to people, the reverse also occurs, mainly in respiratory diseases [42]. In fact, communities close to NHPs have developed simultaneous outbreaks of respiratory diseases caused by different viruses of human origin [43]. For example, both the Ngogo and Kanyawara chimpanzee communities in Kibale National Park, Uganda, developed severe respiratory conditions in December 2016 and January 2017, respectively. Such diagnoses include human metapneumovirus (MPV; *Pneumoviridae*: *Metapneumovirus*) infection, with a mortality of 12.2%, and human respirovirus 3 (HRV3; *Paramyxoviridae*; *Respirovirus*, formerly known as parainfluenza virus 3) infection, without deaths of individuals in the community as a direct result of the infection. In both communities, none of the viruses had been previously diagnosed. Phylogenetic analyses revealed that Ngogo’s MPV and Kanyawara’s HRV3 were closely related to globally circulating human viruses, indicating independent sources for both viruses. However, the origins of infection that caused these outbreaks remain unclear, as these chimpanzee communities usually do not have contact with people, especially tourists. This underscores the importance of adopting “real-time” epidemiologic analyses in mortality episodes in NHPs, for rapid diagnosis and timely adoption of control measures [43].

Another outbreak of severe respiratory diseases in chimpanzees, also in Uganda, resulted in the death of an infant and four adults (mortality of 8.9%). In addition, human rhinovirus C, family *Picornaviridae* [39], was identified. This virus is a significant cause of respiratory tract infection in people, especially in children and adults [44]. In chimpanzees, infection with this pathogen can be associated with other important mortality outbreaks, since although outbreaks of respiratory diseases in these wild primates are common, their causes often remain undiagnosed [39].

Human respiratory syncytial virus (HRSV) is another important respiratory virus, identified as one of the main causes of pediatric mortality in humans [45], which has caused outbreaks in NHPs. In the years 2012 and 2014, respiratory symptoms in the local human population, including coughing, nasal discharge, sneezing, high respiratory rate, and wheezing sounds during exhalation, followed non-lethal outbreaks of respiratory disease in habituated wild western lowland gorillas in the Central African Republic. There were four groups of gorillas with controlled human contact. Human activities such as hunting, collection, fishing, and agriculture are prohibited, but tourism is regulated. From May 2012 to March 2013, mountain gorillas in Rwanda, Uganda, and the Democratic Republic of Congo (DRC) also developed a minimal nasal discharge, sneezing, and intermittent cough resulting from HRSV infection [36].

In the DCR, two outbreaks of respiratory diseases occurred in 2014 and 2015 in two groups of wild bonobos (*Pan paniscus*). Bonobos were not yet habituated in a region that is not protected by law but belongs to local communities that have traditionally not hunted bonobos [38]. However, community members often enter the forest to hunt other animals, collect forest products, or wash their clothes. Presumably, this human use of the forest was sufficient to cause disease spread to the two bonobo groups, which presented respiratory symptoms and nasal discharge. Morbidity ranged from 20% to 30%, and lethality was approximately 10% but these figures may be underestimated, as members of the bonobo groups were not monitored on an individual basis. In these outbreaks, HRSV infection was identified as the cause of pneumonia, in addition to secondary bronchopneumonia, resulting from infection by *Streptococcus pneumoniae*. Aspects of these outbreaks in bonobos speak to the threat of HRSV in other great ape species and other NHPs, since co-infection with other agents (e.g., bacteria) could cause severe problems in primate ecological communities.

**Figure 2:**
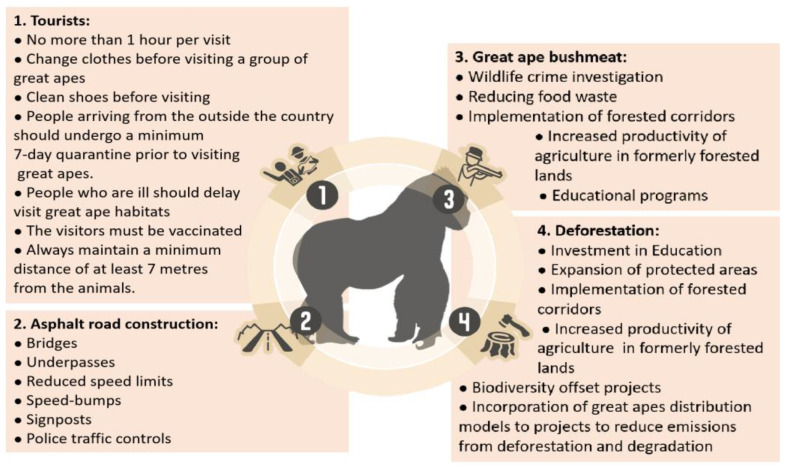
The best practice for the prevention and control of diseases in wild great apes. According to Gilardi et al. [49], Dobrovolski et al. [50], Estrada et al. [51], Tagg et al. [52],Guynup [53], Cibot et al. [54], Kormos et al. [55], and Dickson et al. [56].

These outbreaks of neglected infectious diseases in great apes will continue to occur in Africa unless urgent preventive measures are taken [46,47]; these changes include creating new rules for visiting tourists and tourism operators, public policies for rural development and combating poverty in rural areas, and the use of new technologies to preserve biodiversity. The best practice for the prevention and control of diseases in great apes are exemplified in Figure 2. In addition, disease outbreaks at primate field sites and rural communities must be quickly diagnosed so that effective prevention and control measures can be adopted [48].

## 4. Conclusions

A critical strategic breakthrough is needed in the area of great ape disease prevention. In this context, to prevent the emergence of new diseases, conservation of primate species must be based on the One Health concept. That is, primate conservation efforts include the monitoring of the environment and involve political and social aspects, and anthropological relations in the ape–human interface. 

## Figures and Tables

**Table 1:** Reports of clinical disease in wild great apes due to human viruses during the period 2016–2020.

Pathogen	Great Ape	Year	Reference
HRSV ^1^	mountain gorilla (*Gorilla beringei beringei*)	2020	[36]
	western lowland gorilla (*G. gorilla*)	2016	[37]
	bonobo (*Pan paniscus*)	2018	[38]
HRV-C ^2^	chimpanzee (*Pan troglodytes*)	2018	[39]
HRSV + HMPV ^3^	chimpanzee (*Pan troglodytes*)	2017	[40]
HCoV-OC43 ^4^	chimpanzee (*Pan troglodytes verus*)	2016	[41]

^1^ Human respiratory syncytial virus. ^2^ Human rhinovirus C. ^3^ Human metapneumovirus. ^4^ Human coronavirus OC43.
